# Strategies to Improve Drug Strength in Nasal Preparations for Brain Delivery of Low Aqueous Solubility Drugs

**DOI:** 10.3390/pharmaceutics14030588

**Published:** 2022-03-08

**Authors:** Patrícia C. Pires, Márcio Rodrigues, Gilberto Alves, Adriana O. Santos

**Affiliations:** 1 Faculty of Pharmacy (FFUC-UC), University of Coimbra, Azinhaga de Santa Comba, 3000-548 Coimbra, Portugal; 2 Health Sciences Research Centre (CICS-UBI), University of Beira Interior, Avenida Infante D. Henrique, 6200-506 Covilhã, Portugal; marciorodrigues@fcsaude.ubi.pt (M.R.); gilberto@fcsaude.ubi.pt (G.A.); 3 Faculty of Health Sciences (FCS-UBI), University of Beira Interior, Avenida Infante D. Henrique, 6200-506 Covilhã, Portugal; 4 Center for Potential and Innovation of Natural Resources, Research Unit for Inland Development (CPIRN-UDI-IPG), Polytechnic Institute of Guarda, 6300-559 Guarda, Portugal

**Keywords:** brain delivery, intranasal, nanosystem, nose-to-brain, prodrug, solubilizer

## Abstract

Intranasal administration is a promising route for brain drug delivery. However, it can be difficult to formulate drugs that have low water solubility into high strength intranasal solutions. Hence, the purpose of this work was to review the strategies that have been used to increase drug strength in intranasal liquid formulations. Three main groups of strategies are: the use of solubilizers (change in pH, complexation and the use cosolvents/surfactants); incorporation of the drugs into a carrier nanosystem; modifications of the molecules themselves (use of salts or hydrophilic prodrugs). The use of high amounts of cosolvents and/or surfactants and pH decrease below 4 usually lead to local adverse effects, such as nasal and upper respiratory tract irritation. Cyclodextrins and (many) different carrier nanosystems, on the other hand, could be safer for intranasal administration at reasonably high concentrations, depending on selected excipients and their dose. While added attributes such as enhanced permeation, sustained delivery, or increased direct brain transport could be achieved, a great effort of optimization will be required. On the other hand, hydrophilic prodrugs, whether co-administered with a converting enzyme or not, can be used at very high concentrations, and have resulted in a fast prodrug to parent drug conversion and led to high brain drug levels. Nevertheless, the choice of which strategy to use will always depend on the characteristics of the drug and must be a case-by-case approach.

## 1. Introduction

Intranasal administration is most commonly used for the treatment of local affections, such as nasal congestion, rhinitis or sinusitis, symptoms that are generally related to allergies or upper respiratory tract infections [1]. Nevertheless, having a large surface area-to-volume ratio and high vascularization, the nasal cavity is also favorable for systemic drug absorption and has proven useful for therapeutic systemic effects, having several potential advantages when compared to other routes [2,3]. One of these advantages is its non-invasiveness and ease of administration, hence not requiring trained professionals or in-hospital setting. This feature, together with potentially fast absorption and onset of action, makes intranasal delivery favorable for the management of certain emergency situations such as acute seizure episodes or opioid overdose [4,5]. 

Moreover, the intranasal route has proven to be better accepted by adults and adolescents than the rectal route, and it can also be an alternative for patients for whom the oral and buccal routes are not suitable, such as in conditions associated with vomiting, increased salivation, or inability to swallow. It can also offer an escape from the gastrointestinal tract’s acidic environment and enzymes, and the first-pass hepatic metabolism, being an alternative for drugs with low oral bioavailability. Hence, there are already many intranasal medicinal products on the market for systemic delivery, namely for the treatment of menopausal symptoms, endometriosis or migraine headache, among several other conditions [1,3].

The intranasal route is particularly promising for brain drug delivery, since it makes it possible for drugs to bypass the blood–brain barrier, which has little to no permeability to most of them, and where plenty of molecules can undergo extensive active efflux. Moreover, even in the case of drugs that do cross the blood–brain barrier, it may allow for at least part of the drug to be transported directly from the nasal cavity to the brain, potentially increasing drug bioavailability in the biophase, therefore reducing therapeutic drug doses and minimizing systemic adverse effects [2].

**Figure 1:**
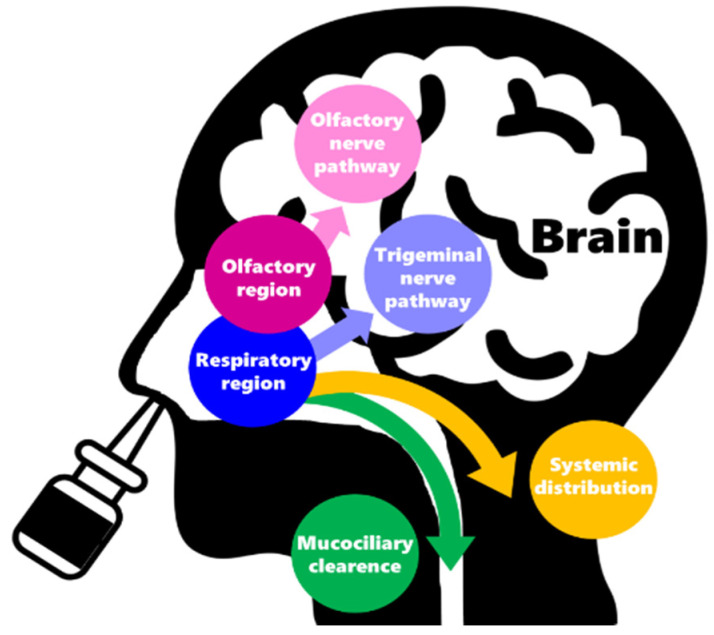
Drug distribution pathways associated with intranasal administration.

Direct nose-to-brain drug transport occurs through neuronal transport or passive diffusion, either from the respiratory or olfactory regions (Figure 1) [3,6,7]. From the olfactory region (smaller surface area), the drugs will reach the brain directly via the olfactory nerve pathway. From the respiratory region (larger surface area), drugs can either diffuse directly to the brain via the trigeminal nerve pathway, enter the systemic circulation, or undergo mucociliary clearance (swallowing). After reaching the systemic circulation, the drugs might still reach the brain indirectly, by crossing the blood–brain barrier (molecules with a lipophilic nature).

Despite its numerous advantages, intranasal delivery also has its limitations, the most important being: the fact that only a low volume can be administered (maximum 150–200 μL in humans), therefore requiring potent drugs or high drug strengths; a short residence time in the nasal cavity due to mucociliary clearance, which can limit the time available for drug absorption to occur; enzymatic degradation and efflux transporters, which could likewise reduce drug absorption [2,3]. Furthermore, it is also very important to carefully consider formulation aspects, since none of the formulation’s components should be irritant to the nasal mucosa, its pH should be similar to the nasal mucosa’s (5.0 to 6.5), and it should be isotonic to slightly hypertonic, all in order to avoid causing sensations of discomfort or toxicity in the nasal epithelium and/or enhanced mucociliary clearance. Moreover, preparation’s viscosity and/or bioadhesiveness should be carefully considered, since a high viscosity/adhesiveness can increase contact time with the nasal mucosa, but if the viscosity is too high the drug diffusion from the formulation might be reduced, which can lead to decreased absorption [1,2,8].

Independently of the administration route, it is difficult to formulate drugs that have low water solubility at high strength without having to use substantial amounts of cosolvents or surfactants, which are potentially toxic excipients. This is an even bigger problem in intranasal delivery, since the drug has to be administered in a small volume, as mentioned before, and thus even higher drug strengths are required [2,3]. 

Hence, the purpose of this work was to review the strategies that have been used to increase drug solubility/strength in formulations intended to be administered intranasally, to treat central nervous system disorders.

## 2. Overview of Intranasal Formulation Strategies for Drugs with Low Aqueous Solubility

Drugs could be intranasally administered simply in the form of dry powders, but the slow dissolution of poorly water-soluble drugs will be a clear limiting factor. The dissolution limitation is the same with liquid suspensions, which are perhaps the most common nasal liquid preparations for drugs with low aqueous solubility, generally intended for a local effect. Nasal sprays of lipophilic corticosteroids (such as budesonide or fluticasone propionate) are examples of such formulations. Nevertheless, the administration of these preparations leads to most of the volume being swallowed, which means that the majority of the drug is not going to have the intended local therapeutic effect. These drugs usually have a lag time of up to several days to start having a therapeutic effect. In fact, even when aiming for a local effect, better results can be obtained by solubilizing the drug. Examples include the solubilization of budesonide in nasal formulations by using Captisol^®^, a proprietary cyclodextrin derivative (sulfobutylether-β-cyclodextrin), or Budesolv, developed using Marinosolv^®^, a proprietary solubilizing technology platform based on plant derived saponins which form a micellar solution [9,10]. 

The development of solid dispersions can be expected to reduce the slow dissolution limitation found in dry powders and suspensions of low water solubility drugs, and examples of these are found in the scientific literature. The budesonide solid dispersion Soluplus^®^, prepared through freeze-drying of a polymer-drug solution, showed faster release compared to both water-based suspension and dry powder commercial products, and higher permeation across a nasal cell model [11]. Cyclodextrin derivatives can also be used in combination to prepare soluble inclusion complexes [12]. However, this review will not focus on these approaches, but rather on liquid preparations (Table 1). 

Alternatively to suspensions and solid dispersions, an emulsion (either liquid or semisolid) could be used, but drug retention in the vehicle/base could originate similar limitations in drug release. Alternatively, the limitations of slow dissolution/release of the drug from suspensions or emulsions could be (at least partially) solved by reducing particle or droplet size to a colloidal/nanometric range, thus increasing the specific surface area, making drug dissolution or diffusion faster. Optionally, other nanometric drug carriers can also be considered, with added functionalities, such as protecting drugs from enzymatic and chemical degradation, increasing transport through biological membranes, and overall promoting brain bioavailability [2,27,28]. Moreover, these nanosystems can have components with permeation enhancing capability, that either act by increasing the nasal membrane’s fluidity by creating temporary hydrophilic pores (due to extracting proteins from it), decreasing the viscosity of the mucous layer, or transiently altering tight junctions [3,29].

Nevertheless, there is no doubt that the simplest and most direct way to deliver the drug readily available for absorption is to formulate it in an aqueous solution. Classical ways to increase drugs’ water solubility are well known and include strategies related to the modification of the chemical entity itself or strategies dependent on formulation excipients. Modification of the drug molecule itself includes salt formation (which can be useful when the chemical entity has an ionizable moiety) and hydrophilic prodrug development (molecule with an additional moiety that has to be metabolized in order to exhibit pharmacological activity), both of which can result in not only higher aqueous solubility but also increased chemical stability [30]. As for strategies concerning formulation excipients, these include pH control (useful with weak acidic or basic drugs), the use of organic solvents (either the change of the solvent to an oil or, preferentially, the use of water-miscible organic solvents in mixture with water), the use of hydrophilic surfactants (above the critical micellar concentration), and the preparation of soluble complexes. In addition, a combination of these strategies can also be employed.

The issue of the formulation having a short residence time in the nasal cavity, and therefore decreasing the time available for drug absorption to occur (and consequently bioavailability), can also be tackled by adding certain components to either nanocarriers or solutions. The inclusion of a mucoadhesive polymer, such as pectin, chitosan, sodium alginate, or certain cellulose derivatives, will help these formulations interact with the nasal mucosa, and thereby retaining the preparation in the nasal cavity for a longer period of time. The use of polymers that increase a formulation’s viscosity (viscosifiers), such as cellulose derivatives, or gelling polymers, such as poloxamers, can also be an efficient strategy in increasing the retention time in the nasal mucosa [2,3,31,32].

In order to help define formulation parameters, including in the case of nanoformulations, bio/chemoinformatics (in silico) tools can be used, with some recent studies having done a good job predicting nose-to-brain transport of antibiotics for the treatment of meningitis, or even for combating COVID-19 [1,2]. Nevertheless, this review will focus on the experimental approach. 

Examples, benefits and limitations of all the mentioned experimental strategies (water-solubilization and colloidal liquid dispersions) in the context of nasal preparations, in particular aiming for nose-to-brain drug delivery, are discussed in the subsequent sections, and summarized in Table 2. Frequently, different strategies can be found combined.

## 3. Use of Excipients for Enhanced Aqueous Solubility 

### 3.1. Adjustment of the pH

The nasal mucosa’s pH is slightly acidic (≈5–6.5), hence a neutral to slightly acidic pH is well tolerated. It would be ideal for this pH range to be sufficient to solubilize any drug molecule, but, if that were the case, the drug would probably not even be considered poorly water soluble. Some drugs require more extreme pH values for solubilization, and a pH below 4 seems to be less well tolerated, as described in the following examples.

K-604 is a selective acyl-coenzyme A:cholesterol acyltransferase-1 inhibitor that blocks cholesterol esterification, which in the brain has been linked to the clearance of amyloid beta peptides and suppression of 24(S)-hydroxycholesterol induced neuronal cell death. Hence, it can have potentially beneficial effects in several neurodegenerative disorders, such as Alzheimer’s disease, also having recently been reported as a promising strategy to treat glioblastoma [13,33]. Nevertheless, alongside having poor blood−brain barrier permeability, its poor water solubility at neutral pH (0.05 mg/mL) makes it hard to formulate at high strength. Yet, since K-604′s solubility increases at a lower pH, Shibuya et al. [13] prepared several solutions containing hydroxycarboxylic acids, either hyaluronic acid, gluconic acid or citric acid, for intranasal administration. These solutions, with pH values ranging from 3.0 to 3.8, were able to solubilize an increased amount of K-604, 10.8 mg/mL, which is 216 times higher than its solubility in purified water. In vivo intranasal administration of these solutions to mice showed enhanced drug delivery efficiency, since the obtained brain and blood area under the drug concentration vs. time curve (AUC) values were approximately between 100 and 211 times higher than those obtained with oral administration. The citric acid solution, having reached the highest brain maximum drug concentration (C_max_), was selected for repeated intranasal administration, to assess therapeutic efficacy (reduction of brain cholesteryl ester levels). However, even though the administration was performed on seven consecutive days, the brain lipid profiles showed that after the first day the brain cholesteryl ester levels had already been reduced up to 94%. Nevertheless, after the 7-day time period, histological evaluations of respiratory and olfactory epithelium of the mice in which the administration was performed showed slight disruption, which could have led to enhanced drug permeability. Hence, although this approach seemed to show high therapeutic promise, its safety profile does not seem ideal, as expected from an acidic formulation.

The development of intranasal benzodiazepine medicines for sedation and seizure control has received a substantial amount of attention. In general, clinical trials show that intranasal benzodiazepines are at least as effective in stopping seizures and preventing their recurrence as the same drugs administered through the intravenous, intramuscular, rectal, or buccal routes [34,35,36,37]. One of the biggest focuses has been on intranasal midazolam, which has a predicted intrinsic water solubility of only 0.00987 mg/mL [38]. Hence, in intravenous administration simple acidified saline solutions (pH 3–4) are used, since midazolam converts to its water-soluble form at this pH [39]. However, lacrimation, throat, and nose burning, and general discomfort are associated with the intranasal administration of these same preparations, which shows that albeit effective, there was still room for improvement regarding formulation safety [4,40].

### 3.2. Cyclodextrins

Cyclodextrins are cyclic oligosaccharides with a central hydrophobic cavity that can form inclusion complexes with hydrophobic drugs. The outer hydrophilic surface, which will be in contact with the external aqueous environment, renders the complex water-soluble, increasing apparent drug solubility [15].

Allopregnanolone (or brexanolone) is a neuroactive steroid (gamma-aminobutyric acid A receptor positive modulator) approved by the FDA for the treatment of postpartum depression in adult females, under the brand name Zulresso^TM^. Allopregnanolone’s predicted water solubility is very low (only 0.00136 mg/mL), but with sulfobutylether-β-cyclodextrin, the solubilizing agent in Zulresso, it was possible to obtain a concentrated solution (5 mg/mL) for intravenous perfusion [41]. An adaptation of this formulation for intranasal administration, an aqueous solution containing 0.9% NaCl and a large amount of sulfobutylether-β-cyclodextrin (40%), reached a drug concentration of 16 mg/mL, and has been shown to provide rapid seizure protection [14].

Curcumin is an extensively studied natural polyphenolic compound, with many known properties, such as antioxidant and anti-inflammatory effects. These properties could be useful for the treatment of many illnesses, such as Alzheimer’s disease [42]. Nevertheless, its poor aqueous solubility (0.00575 mg/mL), high instability under physiological conditions, rapid metabolism, and fast elimination lead to low oral bioavailability and poor tissue distribution. Moreover, it also has very limited permeation through the blood–brain barrier, which further restricts its delivery to the brain [15,43]. To tackle some of these issues, Zhang et al. [15] studied the solubilization of curcumin by hydroxypropyl-β-cyclodextrin (HP-β-CD, 300 mM), which increased from ~1.5 × 10^−4^ mM to ~3 mM (~2000-fold). They also prepared inclusion complexes of curcumin:HP-β-CD, which were lyophilized and resuspended in water at a greater drug strength. These inclusion complexes and which performed better than chitosan-coated poly(lactic-co-glycolic acid) nanoparticles (described in Section 4.2) in protecting the drug from degradation at physiological pH and promoting bioavailability through the intranasal route, having reached much higher brain drug levels than plasma’s, suggesting the existence of a considerable amount of direct transport through the neuronal pathways. However, the administration volume was too large, and the obtained bioavailability was not compared to other routes, which makes it difficult to fully assess the potential of the developed formulation. Moreover, the inclusion complexes did not perform much better than a simpler similar solution, made from curcumin solubilized in DMSO first and then diluted with 0.3 M HP-β-CD. 

As for safety, in general natural and modified cyclodextrins are considered safe, since they are listed as inactive ingredients and accepted as excipients in pharmaceutical products by the FDA. Nevertheless, their safety is dependent on the type of cyclodextrin, their concentration and the administration route. For intranasal administration, preclinical toxicity studies have shown that, in general, a concentration below 10% in the formulation causes low to no local irritation [44].

### 3.3. Cosolvents and Surfactants

As previously mentioned, intranasal benzodiazepines have shown to be at least as effective in stopping seizures and preventing their recurrence as intravenous, intramuscular, rectal, or buccal administrations of the same drugs [34,35,36,37]. Several clinical trials with intranasal administration of benzodiazepines have been undertaken, either using parenteral solutions employing cosolvents, or alternative formulations, including using a liquid surfactant as solvent (Polyoxyl 35 castor oil or Cremophor^®^ EL, nowadays brand name Kolliphor^®^ EL) (reviewed by Pires et al. [4]). Midazolam and diazepam innovative formulations have since reached the market. Nayzilam^®^, a midazolam nasal spray, developed by Proximagen, Ltd. (London, UK), received FDA approval in 2019 for the acute treatment of seizure clusters. However, the drug’s solubilization (at 50 mg/mL) was only achieved by using ethanol, polyethylene glycol-6 methyl ether, polyethylene glycol 400, and propylene glycol [45]. Valtoco^TM^, a diazepam formulation with drug strengths between 50 and 100 mg/mL developed by Neurelis, also reached the market in 2020 for the same indication. Valtoco’s vehicle is composed of vitamin E, ethanol, benzyl alcohol, and n-dodecyl beta-d-maltoside (Intravail^®^—proprietary surfactant used as permeation enhancer) [46,47,48]. Hence, drug solubilization was in both cases performed by adding high amounts of organic cosolvents and/or surfactants to the preparations. Nevertheless, organic solvents have been associated with reports of lacrimation, alteration in taste sensation, rhinorrhea, and burning and general discomfort in the nose and upper respiratory tract to different extents, depending on the exact excipients and their dose [4,40,49,50,51,52,53]. Despite favorable results regarding the approved midazolam and diazepam intranasal formulations, there seems to be scope for improvement regarding formulation tolerability. In fact, side effects could be expected to have a stronger negative impact in treatment compliance in more frequent, non-emergency, treatment regimens.

## 4. Nanometric Dispersions 

### 4.1. Nanosuspensions

Nanosuspensions are dispersions of fine colloidal solid drug particles (<1000 nm, also named drug nanocrystals) in an aqueous vehicle, stabilized by surfactants. They are especially useful if a given molecule has poor solubility in both aqueous and non-aqueous solvents. Nanosuspensions are relatively simple to prepare and have been stated to have several advantages over other nanoformulations, such as controlled drug release, targeted drug delivery, improved bioavailability, reduced dosing frequency, reduced toxicity, and better stability [54,55]. 

Clozapine is an atypical antipsychotic drug frequently used to treat schizophrenia symptoms and acts by blocking dopamine D2 and serotonin 5-HT2 receptors, having few extrapyramidal side effects [56]. Nevertheless, its water solubility is only 0.186 mg/mL [57], which makes strategies to increase its strength in formulation highly relevant. Additionally, its poor dissolution rate, high gastrointestinal degradation and high hepatic first-pass metabolism in oral forms makes it difficult for the drug to reach the brain, and its severe adverse drug reactions (such as agranulocytosis, hepatotoxicity, and cardiotoxicity) lead many patients to discontinue the treatment [56]. Therefore, Patel et al. [56] suggested that exploring a non-invasive route other than oral administration, such as intranasal administration, could be beneficial. Moreover, to tackle clozapine’s solubility problem, a nanosuspension was developed using a combination of (+)-alpha-tocopherol polyethylene glycol 1000 succinate, a surfactant, stabilizer, and permeation enhancer, and polyvinylpyrrolidone K-30, a suspending agent. The obtained formulation drug strength was moderate (0.5 mg/mL), but still more than 42 times higher than its water solubility. The in vivo pharmacokinetic study compared the intranasal administration of the developed nanosuspension and the oral administration of a conventional suspension to rats. Results showed that clozapine brain AUC was significantly higher for the intranasal group (when compared to the oral route), and that the nasal route resulted in a very high brain-to-plasma concentration ratio at 1 h. Aside from having high efficacy in reaching the brain through intranasal administration, the developed nanosuspension also appeared to be safe, since in histopathology studies the nasal mucosa treated with the nanosuspension did not show any signs of epithelial damage. 

Curcumin was also developed as a suspension of nanocrystals, which promoted uptake by olfactory ensheathing cells, a particular type of glial cells that accompany the unmyelinated olfactory axon of receptor neurons (compared to the free curcumin). Final formulation drug strength was 3.42 mg/mL, which is approximately 570 times higher than curcumin’s predicted water solubility. However, the formulation was not evaluated in vivo [16]. 

### 4.2. Polymeric Carrier Nanosystems

Polymeric carrier nanosystems are, as the name implies, nanosystems made of polymers. These polymers can be tailored to adjust surface charge, cargo-loading and cargo-release capability, and surface decoration with specific chemical moieties can prevent the nanosystem’s recognition by the immune system’s cells or reach and attach to a certain therapeutic target. A variety of subtypes can be described, based on either the structure of the nanosystem or the polymer.

Although the classification can differ between authors, polymeric nanoparticles can be considered a subtype of polymeric nanosystems described as matrix-like compact colloidal systems made of water-insoluble polymers, in which drugs are dissolved, entrapped, encapsulated, or attached to. Regardless of having a highly variable size range within the nanometric scale, their surface hydrophobicity and controlled drug release capacity make them promising candidates, having been reported to prolong the extent of therapeutic effect [1,28].

Curcumin has also been encapsulated in chitosan-coated poly(lactic-co-glycolic acid) nanoparticles for intranasal administration by Zhang et al. [15]. Poly(lactic-co-glycolic acid) is a biocompatible and biodegradable copolymer that has been widely used to deliver hydrophobic drugs within nanoparticles. Chitosan was chosen to coat the surface of the nanoparticles since it can transiently open the tight junctions between nasal mucosa epithelial cells to enhance paracellular drug transport to the brain. The developed formulation was administered intranasally to mice, however, it had an inferior performance when compared to the cyclodextrin inclusion complexes described in Section 3.2. 

Polymeric micelles, being another subtype of polymeric nanosystems, are made of polymers as well, but their composition differs from polymeric nanoparticles in the way that they are made of amphiphilic block copolymers that spontaneously self-assemble, forming a hydrophobic core and a hydrophilic corona. Because of these structural characteristics, the polymeric micelles’ core can solubilize and incorporate lipophilic drugs, and the hydrophilic corona will be a stabilizing interface between the hydrophobic core and the external aqueous environment, making it a nanosystem with high kinetic stability [58,59,60].

Baicalein is a plant derived flavonoid compound with antioxidant and anti-inflammatory properties that have been associated with neuroprotective effects. Hence, it could be potentially beneficial in the treatment of disorders such as Alzheimer’s disease, in which it has been known to inhibit Tau protein assembly and aggregate formation (which is related to intra-neuronal functional loss), or Parkinson’s disease, in which it has been reported to reduce several pro-inflammatory cytokines’ production and overall suppress neuroinflammatory pathways [61,62]. Nevertheless, although it is a molecule with high permeability through the biological barriers, baicalein has poor water solubility (0.153 mg/mL) [63]. Moreover, after oral administration this drug is extensively metabolized in the intestine and liver. In order to try to formulate baicalein at a high strength, and protect it from metabolism, Zhang et al. [17] developed poly(ethylene glycol)-block-poly(D,L-lactide) micelles, a biodegradable and nontoxic amphiphilic di-block polymer. In these micelles, baicalein was encapsulated into the poly(D,L-lactide) core (hydrophobic), and the poly(ethylene glycol) coating (hydrophilic) allowed the formation of micellar structures in aqueous media. Intranasal administration of an aerosol of these micelles to mice led to brain C_max_ and AUC values that were 1.44 and 1.50 times higher than those obtained with the oral administration of a baicalein coarse powder suspension. Therefore, these results seem to show that the intranasal administration of the developed baicalein micelles could facilitate brain drug delivery, when compared with oral dosing. Furthermore, toxicity studies showed that the micelles had much less cytotoxicity in neuronal cells than a drug solution, and a 5-day continuous daily intranasal administration study showed that the nasal mucosa maintained its integrity, with no evidence of tissue necrosis or inflammation. This suggests that the developed formulation could be safe for chronic administration.

### 4.3. Solid Lipid Nanoparticles and Nanostructured Lipid Carriers

Lipid-based matrix-type nanoparticles have long been regarded as having a high safety profile due to their biocompatible and biodegradable lipid composition. These matrices have also been claimed to improve the stability of labile molecules, consequently improving their bioavailability, while guaranteeing a controlled release profile [20,64,65]. Additionally, solid lipid nanoparticles, made of solid lipids dispersed in water or an aqueous surfactant solution, have been reported to allow increased nasal retention (due to occlusive effect and mucous membrane adhesion) [64,65].

The natural compound geraniol (GER) is known to promote the survival of dopaminergic neurons by enhancing the production of antioxidant enzymes, reducing apoptotic marker expression, and increasing the production of neurotrophic factors. On the other hand, the secondary bile acid ursodeoxycholic acid (UDCA) has shown to have a protective effect against mitochondria-dependent programmed cell death in parkinsonian patients. In order to combine the neuroprotective, anti-inflammatory and antioxidant properties of these two molecules to treat Parkinson’s disease, Junior et al. [19] synthesized a prodrug containing both. Thus, they produced GER-UDCA, a highly hydrophobic conjugate, which led to an extremely low water solubility molecule (0.00023 mg/mL). Hence, the authors decided to formulate this conjugate into solid lipid nanoparticles containing the lipid glyceryl dibehenate (Compritol^®^ ATO 888), and the hydrophobic surfactant sorbitan trioleate (Span^®^ 85). The in vivo intranasal administration of these solid lipid nanoparticles to rats showed a selective uptake of GER-UDCA to the cerebrospinal fluid, suggesting the existence of a direct nose-to-brain pathway. Furthermore, the solid lipid nanoparticles seemed to prolong and enhance brain drug levels, when compared to the oral route. Moreover, the histopathological evaluation of rat nasal mucosa showed no signs of damage after the nanosystem’s administration, whereas GER alone (glycerol and water dispersion) caused high levels of irritation. Therefore, intranasal administration of the developed GER-UDCA solid lipid nanoparticles demonstrated the ability to induce nose-to-brain conjugate permeation, without damaging the nasal mucosa.

Nanostructured lipid carriers are lipid nanoparticles made of a mixture of solid and liquid lipids, which create an imperfect and disordered crystal matrix within which hydrophobic drugs can be better accommodated than in solid matrices. They have been stated to allow rapid uptake, high drug loading, and very good long-term stability [20,66].

There are many diseases that have been described to benefit from the pharmacological manipulation of the endocannabinoid signaling, including several that affect the central nervous system (movement and anxiety disorders, cognitive dysfunction, neuropathic pain, etc.). However, cannabinoid drugs are known to have low solubility in aqueous media. Esposito et al. [20] used rimonabant (water solubility 0.002 mg/mL) [67] as a model cannabinoid antagonist to try to address this issue and formulated it within nanostructured lipid carriers for intranasal administration. These carriers contained a lipid mixture of tristearin and triglyceride (Miglyol 812 N), and the surfactant polysorbate 80, which has been described to increase brain targeting and inhibit the P-glycoprotein (existing in both the nasal cavity and the blood–brain barrier). The achieved drug strength, 2.147 mg/mL, was more than 1000 times higher than rimonabant’s water solubility. The intranasal administration of these nanostructured lipid carriers to rats led to high brain/blood ratios, and these ratios were also higher than the obtained from the intranasal administration of a rimonabant solution (made of polyethylene glycol, polysorbate 80, and saline solution at 90%). Since polysorbate 80 was present in both the nanocarrier and the solution, it appeared that the nanosystem itself did in fact have an additional role in promoting brain targeting.

Another promising nanostructured lipid carrier system was the one developed by Madane et al. [18], containing curcumin, for the treatment of brain cancer. The composition included Precirol^®^ ATO5 (solid matrix), Capmul^®^ MCM (liquid lipid), Tween^®^ 80 (surfactant), and soya lecithin (stabilizer). The obtained particle size (146.8 nm) and PDI (0.18) values were good, and the nanostructured lipid carrier had increased cytotoxicity in astrocytoma-glioblastoma when compared to a drug suspension. In histopathologic studies, no change in sheep nasal mucosa was observed 6 h after applying curcumin-loaded nanostructured lipid carrier system. Moreover, in vivo pharmacokinetic studies showed that the intranasal administration of the developed nanosystem was possible at a high dose (50 mg), resuspended in 0.1 mL, and led to higher brain drug levels (C_max_ of 86,201 ± 8182  ng/g at 120 min) when compared to the drug suspension (C_max_ of 54,321 ±  2098.8 ng/g at 180 min).

### 4.4. Liposomes and Liposome-Related Vesicular Nanosystems

Liposomes are biocompatible and biodegradable vesicles made of phospholipid and cholesterol bilayers. One or more aqueous compartments can exist depending on how many bilayers they have [1,27]. Several variations of these particles have been developed, with one of them being transfersomes. Transfersomes have membrane incorporated edge activators in their lipidic bilayers, which gives them high flexibility and deformability [68]. These edge activators are usually single-chain surfactants, which makes it easier for these vesicles to change their shape and squeeze between cells, enhancing their mucosal permeation. They have also been reported to enhance nasal mucosa permeation by opening new pores through the paracellular tight junctions [21,22].

Flibanserin is a non-hormonal drug used for the treatment of hypoactive sexual appetite disorder in women, acting by decreasing serotonin and increasing dopamine and norepinephrine levels [21]. Its oral administration leads to reduced bioavailability, which can be associated with hepatic first-pass metabolism, but also with its low water solubility (0.178 mg/mL) [69]. Ahmed et al. [21] decided to formulate flibanserin into transfersomes, containing the phospholipid phosphatidylcholine, and the hydrophobic surfactants Span^®^ 65 and 80 (since Spans—sorbitane esters—are common and efficient edge activators). The authors loaded a hydroxypropyl methylcellulose (HPMC) matrix with the transfersomes, making a flibanserin transfersome loaded HPMC based hydrogel. By using HPMC, a mucoadhesive and viscosifying polymer, the purpose was also to extend the residence time of the formulation in the nasal cavity and have a sustained drug release. The achieved drug strength was 10 mg/mL, which is a more than 56-fold increase when compared to the drug’s water solubility. The in vivo pharmacokinetic study compared the intranasal administration of the transfersome hydrogel with the administration of a simple hydrogel, through the same route, to rats. The transfersome hydrogel led to brain C_max_ and AUC values that were twice as high (when compared to the simple hydrogel), suggesting an enhanced brain delivery due to the nanosystem. In the authors’ opinion, this could be attributed to the synergistic effect of the flexibility and deformability of the transfersomes, and the permeation enhancing capabilities of the surfactants. Furthermore, histopathological evaluation of the nasal tissues showed no signs of damage or inflammation, which could be interpreted as the developed formulation being safe.

Resveratrol is an antioxidant and anti-inflammatory plant derived polyphenol, with a wide range of associated possible therapeutic effects. It has been pointed out as potentially efficient in the treatment and prevention of many neurodegenerative disorders, such as Alzheimer’s disease, in which it has been shown to reduce inflammatory cytokine release, improve mitochondrial energetic function and enhance Aβ-peptide clearance [70]. Yet, its application remains limited by several issues, such as a short biological half-life, which is a consequence of extensive intestinal and hepatic metabolism, leading to it having a very low oral bioavailability [22]. Moreover, it has poor aqueous solubility (0.0688 mg/mL) [71], which makes it difficult to formulate at high strength. To tackle this issue, Salem et al. [22] formulated resveratrol into transfersomes, containing Cremophor^®^ RH 40, ethanol and soya lecithin, and put them into a hydrogel, having Poloxamer 407 and Carbopol^®^ 934 in its composition. Although having other functions within the formulation (cosolvent, and mucoadhesive and gelling polymer, respectively), ethanol and Carbopol were also included to serve as permeation enhancers. The in vivo pharmacokinetic study compared the intranasal administration of the developed transfersomes hydrogel with the oral administration of a resveratrol oral suspension to rats. Although brain drug levels were not determined, the results showed that the resveratrol concentration in plasma after intranasal administration of the transfersome hydrogel was significantly higher at all time points, when compared to the oral suspension, with plasma C_max_ increasing by 2-fold and AUC by more than 20-fold. Moreover, the transfersome hydrogel led to sustained plasma levels, with the drug being quantifiable up to 24 h after the intranasal administration was performed. Additionally, the histopathological examination of the rats’ nasal mucosa showed no signs of severe tissue degeneration.

### 4.5. Nanometric Emulsions

Nanometric emulsions are colloidal liquid-in-liquid dispersions that have the advantage of being quite easy to prepare, since certain formulas emulsify spontaneously just by adding the aqueous phase component to the mixture of oil and surfactants in the right proportions [1]. These nanosystems can be separated into two main types: nanoemulsions and microemulsions. Although it is not easy to distinguish between them, with opinions and definitions varying within the scientific literature, microemulsions are thermodynamically stable systems, and nanoemulsions are not, having, however, a relatively high kinetic stability [72,73,74]. Moreover, in general, microemulsions are considered to have a smaller droplet size range (10–100 nm) than nanoemulsions (20–200 nm). When compared to liquid macroemulsions, nanometric emulsions have a higher surface area and free energy, and increased physical stability, hence potentially having a longer shelf life [75]. Their lipophilic nature, good permeability, and solubilizing effect make them promising systems for the incorporation of liposoluble drugs [76]. Indeed, there are innumerous examples of intranasal delivery of nanometric emulsions containing different drugs, either plain or containing a mucoadhesive and/or viscosifying agent to increase the formulations’ retention in the nasal mucosa [28]. Another strategy to increase this retention would be to promote in situ gelling by adding, for example, a thermosensitive polymer to the nano or microemulsion’s external phase, which when in contact with the higher temperature of the nasal mucosa will increase the formulations viscosity, creating nanoemulgels. These nanoemulgels combine the properties of both nanoemulsions and gels: increased stability and drug solubility (nanoemulsions), and increased viscosity with potentially enhanced retention times (gels) [77]. It is also possible to encapsulate or coat the nanodroplets with a polymer shell, such as in the production of “amylolipid nanovesicles” described ahead in Section 4.6 [24].

Another curcumin formulation, in this case a microemulsion, was developed by Shinde et al. [23], for the treatment of brain cancer. Obtained drug strength was 5 mg/mL, which is 869 times higher than curcumin’s predicted water solubility. The developed formulations had Capmul^®^ MCM, with or without docosahexaenoic acid-rich oil, in their composition, as well as Tween^®^ 80, ethanol and water. Histopathological studies in rats confirmed safety of the microemulsions (blank and containing curcumin) on the nasal mucosa and brain even after the intranasal administration for 14 days. However, the developed formulations were cytotoxic in a concentration dependent manner to human glioblastoma cells and inclusively formulations containing not only curcumin, but also docosahexaenoic acid-rich oil, showed higher cytotoxicity, probably due to a synergistic anticancer activity. In in vivo pharmacokinetic studies, the developed microemulsions led to a higher brain drug concentration when compared to a curcumin solution, with the microemulsion containing Capmul^®^ MCM + docosahexaenoic acid-rich oil performing better than the one having Capmul^®^ MCM only, which suggests that this oil has drug transport enhancing abilities, either by enabling nose-to-brain drug transport, and/or by enhancing drug permeation. Additionally, the intranasal administration of these same formulations led to higher brain drug levels than the intravenous route.

Safety considerations for nanometric emulsions are similar to what was previously discussed for the solubilization by use of surfactants and cosolvents (Section 3.3), since both of these classes of excipients are typically used in nano and microemulsions in addition to oils. Therefore, the choice of excipients should be performed considering the solubilizing power for a specific drug, physical aspects of emulsion formation, and safety issues, also considering of course the desired concentration/dose of administration. While the safety of most of these excipients is well established for cutaneous, oral, or intravenous/parenteral administration, their safety for the intranasal route is not as well-known. More studies should be studied with excised tissues are useful to obtain information on permeation, metabolism, efflux, and toxicity.

### 4.6. Polymer-Coated Nanometric Emulsions

An interesting novel system encapsulating curcumin, which added novel functionality to nanometric emulsions, were the curcumin “amylolipid nanovesicles” developed by Sintov et al. [24]. This nanovesicles with curcumin were prepared by using a microemulsion as the precursor nanosystem, consisting of polyoxyl 40 hydrogenated castor oil, cocoa butter (theobroma oil), tetraglycol, and glyceryl oleate. After addition of starch, a crosslinking reaction was promoted between starch and divanillin in the aqueous phase of the water-in-oil microemulsion or in the aqueous channels formed in the bicontinuous microemulsion. These systems were named “amylolipid nanovesicles” and had mean size between 130 and 150 nm and polydispersity index between 0.11–0.20. The optimized formulation (with 2% crosslinked starch) of curcumin-loaded amylolipid nanovesicles at a dose of 160 μg/kg was tested by intranasal route in a pharmacokinetic study in rats. Brain and plasma levels were 141.5 ng/g and 11.9 ng/mL, respectively, 1 h after administration. These concentrations were significantly higher than those achieved by the intravenous route at the same dose, for which curcumin was not detected in the brain, and the plasma level was approximately one half of the level achieved after intranasal administration. This work clearly illustrates that the success of nose-to-brain drug delivery is supported not only by the increase in drug solubility, but also on promotion of efficient direct transport to the brain. In this regard, a lot of work can still be performed in order to fully understand how to better explore this route.

## 5. Drug Molecule Modification

Salt formation is a very common method of increasing water solubility and dissolution rates of acidic and basic drugs [78], such that most drugs are usually already obtained/prepared as salts. However, this must be associated with pH control, since the salt form of an acidic drug might originate a basic pH, which is not compatible with intranasal administration, or require pH adjustment for maximum solubility. Nevertheless, many times this will not be enough to achieve a formulation drug strength that is high enough for intranasal delivery. For example, phenytoin sodium still has a reduced solubility in acidic and neutral pH, and the parenteral solution at 50 mg/g is only possible to obtain by combining pH > 10 and propylene glycol.

Prodrugs are pharmacologically inactive molecules that can be metabolized to originate their active counterpart, usually by enzymatic cleavage of one or more promoieties. Prodrug development is a strategy used to overcome several problems that can be associated with active molecules, such as instability, poor absorption or distribution, toxicity, and poor solubility [79].

Phenytoin is an antiepileptic drug that acts by blocking voltage-gated sodium channels, thereby inhibiting the positive feedback loop that results in neuronal propagation of action potentials. Nevertheless, it has low aqueous solubility (0.0711 mg/mL), which makes it difficult to formulate at high strength [80]. On the contrary, its hydrophilic phosphate ester prodrug, fosphenytoin, is highly soluble in water, especially in the form of disodium salt. Although one could think that it would be likely for fosphenytoin’s anionic nature to hinder its nasal absorption, Antunes Viegas et al. [81] demonstrated that the nasal mucosa has phosphatase activity, promoting the in-situ bioconversion of fosphenytoin to phenytoin. Moreover, in addition to the conversion to phenytoin and subsequent permeation, a small part of fosphenytoin could permeate an ex vivo model of porcine nasal mucosa in prodrug form. 

Having these results in mind, Pires et al. [25] developed simple aqueous-based formulations containing the prodrug fosphenytoin, the mucoadhesive and viscosifying polymer HPMC, and albumin to increase nose-to-brain transport. The achieved drug strength for an isotonic solution was around 34.8 mg/mL of phenytoin equivalents (50 mg/mL of fosphenytoin), which is 489 times higher than phenytoin’s aqueous solubility. Moreover, this was achieved while using very safe excipients only, and also safe levels of osmolality. In vivo pharmacokinetic results showed that, despite no fosphenytoin being detected in neither mouse blood nor brain (fast bioconversion), the intranasal administration of fosphenytoin led to high brain phenytoin levels. Although the intravenous administration of a fosphenytoin solution was faster in making the drug reach the brain, which could be due to the need of prodrug conversion occurring before drug diffusion through the nasal mucosa, the obtained brain C_max_ values were similar between intranasal and intravenous administrations, which suggests similar efficacy (albeit delayed). Moreover, blood C_max_ was considerably higher for the intravenous administration, which suggests that the intranasal route could be safer, because lower systemic drug levels could lead to a decrease in systemic side effects. Additionally, the presence of albumin in the formulation prolonged phenytoin’s brain drug levels, which led to a higher brain AUC than that obtained with the intravenous route. Moreover, it is important to add that even after a small single dose administration half of the lower limit of mice’s therapeutic level was already reached.

Diazepam is another anticonvulsant drug, acting by indirectly enhancing gamma-aminobutyric acid inhibitory effects in neuronal excitability [82]. Having poor water solubility (0.05 mg/mL), and as happens with other benzodiazepines, diazepam requires cosolvents or other excipients to improve its strength in formulation, which can be highly irritating to the nasal mucosa. To solve this problem, Rautiola et al. [26] decided to use diazepam’s water-soluble lysine prodrug, avizafone, and co-administer it with its converting enzyme, aminopeptidase B. The in vivo pharmacokinetic results showed that intranasal avizafone + enzyme resulted in rapid absorption of diazepam, with high brain and blood levels. Nevertheless, there was no evidence of direct nose-to-brain transport, and hence most of the drug probably reached the systemic circulation before passing the blood–brain barrier and indirectly reaching the brain. Still, this prodrug/enzyme combination in an aqueous vehicle was a successful strategy to formulate diazepam without the use of potentially toxic excipients, being administered through a non-invasive route, and with efficacy in making the drug reach the brain. Moreover, histological analysis of the nasal tissues post-administration showed no inflammatory signs, with very few minimal to mild lesions, and hence the strategy was considered safe.

## 6. Final Remarks

We reviewed the strategies that can be used to increase the drug strength of synthetic or plant derived low molecular weight drugs, with an already established indication or potential for treating several neurological and psychiatric diseases. The strategies can be many, but not all are equally adequate for a specific route, namely intranasal administration. 

We described different strategies, reported by different authors for the same drugs. However, it is not easy to compare them. For example, curcumin has been subjected to different encapsulation/solubilization strategies, in particular, as cited above, complexation with cyclodextrins and incorporation into different nanosystems (nanosuspensions, solid lipid nanoparticles, nanostructured lipid carriers, nanometric emulsions, and polymer-coated nanometric emulsions) resulting, in general, in a (variable) increase in drug strength. However, other determinants (administration dose/volume or direct nose-to-brain transport) can influence brain levels significantly, which cannot be directly compared. For midazolam, cosolvents and surfactants resulted in a commercially available formulation (Valtoco^TM^), but the strategy to change the pH to more acidic values was also evaluated, showing tolerability issues. Regarding diazepam, cosolvents and surfactants were employed, and, also, resulted in a commercially available formulation (Nayzilam^®^), but a salt and a hydrophilic prodrug strategy were also evaluated. The prodrug resulted in higher brain and blood concentrations in rats, thus proving to be a successful strategy to formulate diazepam without the use of potentially toxic excipients. 

In fact, the strategy of decreasing the formulation’s pH below 4 (required to ionize certain drugs) appeared to disrupt the nasal epithelium and cause significant nasal discomfort, which makes it not safe for intranasal delivery. Cyclodextrins (in particular, the most water-soluble derivatives) seem to be safe—the question is whether a sufficiently high drug strength can be obtained, which must be evaluated case-by-case. Cosolvents and surfactants’ solubilization capacity vs. tolerability profile can vary substantially, depending on the administered dose of the formulation and these excipients’ concentration in the preparations themselves. Even some of these excipients that are safely used in parenteral formulations appear to be toxic or poorly tolerated in intranasal delivery. On the other hand, in general, nanoformulations seemed to be safer, showing little to no signs of nasal epithelial damage or inflammation, and cytotoxicity in neuronal cells being low or inexistent. 

Nanosuspensions, polymeric nanoparticles, polymeric micelles, solid lipid nanoparticles, nanostructured lipid carriers, transfersomes, and nanometric emulsions all succeeded in increasing several drugs’ strength in formulation, reaching up to 1000-fold when compared to their water solubility. Moreover, overall, these formulations led to high brain drug levels, with some also reaching high brain-to-plasma ratio values, which could indicate direct nose-to-brain drug transport. Additionally, when compared to other administration routes, such as oral or intravenous, these intranasal formulations were more effective at achieving and/or prolonging brain drug levels. Furthermore, the studies that had pharmacodynamic evaluation concluded that the developed nanosystems were in fact therapeutically effective.

On the other hand, hydrophilic prodrugs, whether co-administered with a converting enzyme or not, resulted in a fast prodrug to parent drug conversion, and led to high brain drug levels, although there was no sign of nose-to-brain transport. Nevertheless, it was still a successful strategy in formulating low solubility drugs without the use of potentially toxic excipients.

It is hard to say what might be the best strategy for the future. The intranasal administration of either a nanosystem or a hydrophilic prodrug seems to be effective in increasing formulation drug strength and making the drug reach the brain, while also being seemingly safe for the nasal mucosa. However, considering the perspective of the pharmaceutical industry, nanometric formulations might not be an alluring option, since they can be overall more expensive to produce (when compared to more simple formulations), and there might be a significant difficulty with their scale-up process. Hence, since the use of cosolvents and surfactants has led to formulations that have already reached the market, this might be, for now, the most promising strategy for intranasal delivery, given a careful consideration on choosing the safest excipients, or considering safe limits. Nevertheless, the choice of which strategy should be applied will always depend on the characteristics of the drug, and therefore a case-by-case approach should be considered.

## Figures and Tables

**Table 1:** Formulation strategies for increasing drug strength of low water solubility drugs in liquid nasal preparations.

Global Strategy	Formulation Strategy	Drug	Approximate Water Solubility (mg/mL)	Achieved Drug Strength ^1^ (mg/mL)	Drug Product or Bibliographic Reference
Use of solubilizers	Change in pH (acidification)	K-604	0.05	10.8	[13]
		Midazolam	0.01	5	Midazolam injection USP
	Complexation (cyclodextrins)	Allopregnanolone	0.001	16	[14]
		Curcumin	0.006	~3	[15]
	Cosolvents and surfactants	Midazolam	0.01	50	Nayzilam^®^
		Diazepam	0.05	50–100	Valtoco^TM^
Nanosuspensions and incorporation into carrier nanosystems	Nanosuspensions	Curcumin	0.006	3.42	[16]
	Polymeric nanosystems	Curcumin	0.006	~1.5	[15]
		Baicalein	0.2	0.8 ^2^	[17]
	Solid lipid nanoparticles and nanostructured lipid carriers	Curcumin	0.006	500	[18]
		Geraniol- ursodeoxycholic acid conjugate	0.0002	~4.5	[19]
		Rimonabant	0.002	~2	[20]
	Liposomes and related vesicular nanosystems	Flibanserin	0.2	10	[21]
		Resveratrol	0.07	NR	[22]
	Nanometric emulsions ^3^	Curcumin	0.006	5	[23]
	Polymer-coated nanometric emulsions ^4^	Curcumin	0.006	1.9	[24]
Drug molecule modification	Salts and hydrophilic prodrugs	Phenytoin (used as fosphenytoin)	0.07	34.8 (equivalent to 50 mg/mL fosphenytoin)	[25]
		Diazepam (avizafone)	0.05	Up to the equivalent of ~13.5 mg/mL of diazepam	[26]

^1^ In the case lyophilized systems, it was considered, sometimes estimated, the strength of the liquid suspension used in vivo; ^2^ concentration after resuspension of the nanoparticles for nebulization (inhalation during 20 min); ^3^ microemulsions and nanoemulsions; ^4^ the one cited here was named by the authors as “amylolipid nanovesicles”; NR—not reported for the final formulation.

**Table 2:** Advantages and limitations of formulation strategies for increasing drug strength of low water solubility drugs in liquid nasal preparations.

Global Strategy	Formulation Strategy	Advantages	Limitations
Use of solubilizers	Change in pH (acidification)	Increased drug solubility	Irritation of the nose and upper respiratory tract
	Complexation (cyclodextrins)	Increased drug solubility, protection, and permeation	Safety is dependent on the type of cyclodextrin, their concentration, and the administration route
	Cosolvents and surfactants	Increased drug solubility and permeation	Irritation of the nose and upper respiratory tract
Nanosuspensions and incorporation into carrier nanosystems	Nanosuspensions	Increased drug strength, simplicity of preparation, controlled drug release, and reduced toxicity	Physical instability and drug precipitation
	Polymeric nanosystems	Increased drug strength, controlled drug release, targeted drug delivery, and prolonged therapeutic effect	Physical instability, low drug loading, and excipients not biocompatible
	Solid lipid nanoparticles and nanostructured lipid carriers	Increased drug strength, high safety (biocompatible), and controlled release profile	Physical instability and low drug loading
	Liposomes and related vesicular nanosystems	Increased drug strength, high safety (biocompatible), and enhanced permeation	Physical instability and low drug loading
	Nanometric emulsions	Increased drug solubilization, easy preparation (some), and enhanced permeability	Physical instability and low drug solubilization
Drug molecule modification	Salts and hydrophilic prodrugs	Increased drug solubility and safety	Might not be enough to increase drug strength (has to be joined by other strategies)

## Data Availability

Not applicable.
